# Detection of Pear Quality Using Hyperspectral Imaging Technology and Machine Learning Analysis

**DOI:** 10.3390/foods13233956

**Published:** 2024-12-08

**Authors:** Zishen Zhang, Hong Cheng, Meiyu Chen, Lixin Zhang, Yudou Cheng, Wenjuan Geng, Junfeng Guan

**Affiliations:** 1 College of Horticulture, Xinjiang Agricultural University, Urumqi 830052, China; zhangzishen2@163.com; 2 Institute of Biotechnology and Food Science, Hebei Academy of Agricultural and Forestry Sciences, Shijiazhuang 050051, China; chenghonghappyok@163.com (H.C.); cmy20000128@126.com (M.C.); feiliuzhao@163.com (L.Z.); chengyudouyn@163.com (Y.C.); 3 Hebei Key Laboratory of Plant Genetic Engineering, Shijiazhuang 050051, China; 4 College of Life Science and Food Engineering, Hebei University of Engineering, Handan 056000, China

**Keywords:** hyperspectral imaging, pear, non-destructive detection, machine learning model, prediction model

## Abstract

The non-destructive detection of fruit quality is indispensable in the agricultural and food industries. This study aimed to explore the application of hyperspectral imaging (HSI) technology, combined with machine learning, for a quality assessment of pears, so as to provide an efficient technical method. Six varieties of pears were used for inspection, including ‘Sucui No.1’, ‘Zaojinxiang’, ‘Huangguan’, ‘Akizuki’, ‘Yali’, and ‘Hongli No.1’. Spectral data within the 398~1004 nm wavelength range were analyzed to compare the predictive performance of the Least Squares Support Vector Machine (LS-SVM) models on various quality parameters, using different preprocessing methods and the selected feature wavelengths. The results indicated that the combination of Fast Detrend-Standard Normal Variate (FD-SNV) preprocessing and Competitive Adaptive Reweighted Sampling (CARS)-selected feature wavelengths yielded the best improvement in model predictive ability for forecasting key quality parameters such as firmness, soluble solids content (SSC), pH, color, and maturity degree. They could enhance the predictive capability and reduce computational complexity. Furthermore, in order to construct a quality prediction model, integrating hyperspectral data from six pear varieties resulted in an *RPD* (Ratio of Performance to Deviation) exceeding 2.0 for all the quality parameters, indicating that increasing the fruit sample size and variety number further strengthened the robustness of the model. The Backpropagation Neural Network (BPNN) model could accurately distinguish six distinct pear varieties, achieving prediction accuracies of above 99% for both the calibration and test sets. In summary, the combination of HSI and machine learning models enabled an efficient, rapid, and non-destructive detection of pear quality and provided a practical value for quality control and the commercial processing of pears.

## 1. Introduction

Fruit quality is a critical factor influencing market value and consumer satisfaction, and it occupies a vital role in global agriculture and the food industry [1]. To ensure fruit quality and safety, there is an increasing demand for rapid and accurate detection technologies. Traditionally, fruit quality assessment relies on precise but destructive, time-consuming, and complex physical and chemical methods that are ill-suited for rapid testing and large-scale applications [2]. As a result, conventional methods struggle to meet the modern demands of fruit quality monitoring.

With advancements in detection technology, hyperspectral imaging (HSI) has emerged as an effective tool for assessing fruit quality due to its non-destructive nature and being extremely precise [3]. HSI allows for the simultaneous detection of various fruit quality parameters, including color, sugar content, and acidity. It can make measurements more precise and also captures subtle variations within samples, making it particularly suitable for complex and heterogeneous fruit samples [4,5,6]. Furthermore, HSI leverages multi-wavelength spectral data in combination with advanced machine learning algorithms to provide superior predictive accuracy and a more comprehensive chemical composition analysis. This enables the holistic and real-time assessment of fruit quality [7]. Weng et al. utilized HSI to predict the quality of strawberries at different storage durations. Their results indicated that partial least squares regression (PLSR) provided the highest predictive accuracy for soluble solids content (SSC) and vitamin C (Vc), while local weighted regression (LWR) was the most effective for predicting the pH [8]. Gao et al. applied HSI to predict the SSC of grapes, and after performing dimensionality reduction using principal component analysis (PCA), the PLSR model demonstrated outstanding results. The calibration and prediction correlation coefficients (*Rc* and *Rp*) reached values of 0.977 and 0.976, respectively, indicating a superior performance [9]. Che et al. employed near-infrared spectroscopy in conjunction with machine learning methods to predict the internal quality of Korla pears. The study demonstrated the feasibility of integrating near-infrared spectroscopy with machine learning techniques for the non-destructive assessment of Korla pears [10]. Zhang et al. demonstrated that HSI technology facilitates accurate and non-destructive detection of the sugar content in ‘Dangshan’ pears [11]. Liu et al. successfully classified lychee varieties, achieving great accuracy, particularly with a support vector machine (SVM) model, which reached 100% accuracy in the calibration set and 87.81% in the prediction set [12].

Machine learning is a computational approach that involves the use of algorithms to analyze large datasets and build predictive models [13]. During the modeling process, the data needed to be preprocessed and feature wavelength selection to improve the accuracy and robustness of the model prediction [14]. Khort et al. combined hyperspectral imaging with machine learning to efficiently detect and classify apple disease lesions, significantly enhancing the precision and efficiency of automated fruit sorting systems [15]. Apostolopoulos et al. developed a machine learning model based on Vision Transformers (VIT) to assess fruit quality by analyzing deep image features, achieving great accuracy in classifying various fruit types [16].

To systematically investigate the effectiveness of HSI in predicting multiple quality indicators of different pear varieties, this work focused on six varieties of pears to apply HSI technology to non-invasive testing to assess the potential in evaluating the quality of the pears. The study employed the LS-SVM model based on 398~1004 nm spectra to examine the detection accuracy by selecting a preprocessing method and feature wavelength. Additionally, the fruit sample size and the number of pear varieties were expanded to boost the accuracy and generalization of the model. A classification model is developed to classify six varieties of pears, exploring the feasibility of using HSI for quality evaluation.

## 2. Materials and Methods

### 2.1. Sample Preparation

The materials included six varieties of pears: ‘Sucui No.1’, ‘Huangguan,’ ‘Zaojinxiang’, ‘Akizuki’, ‘Yali’, and ‘Hongli No.1’. In this experiment, HSI data under 398~1004 nm from 200 fruit from each variety were collected, totaling 1200 samples. The relevant information is presented in Table 1 below:

### 2.2. Hyperspectral Imaging System

The spectral imaging system consisted of a hyperspectral camera (SPECIM FX 10, Spectral Imaging Ltd., Oulu, Finland) and a spectral imaging workstation (Lab scanner 40 × 20, Spectral Imaging Ltd., Oulu, Finland) with a spatial resolution of 1024 pixels (pixel size of 8 × 8 nm) and a spectral resolution of 448 bands, covering a spectral range of 398~1004 nm. In addition, the system was equipped with a computer featuring dedicated software that allows setting all system parameters according to experimental needs (Figure S1). The acquisition speed for full-band imaging was 330 fps, and the spectral resolution was 5.5 nm. The illumination system used a dual-lighting scheme of six 20 W halogen lamps to reduce interference from external light during image acquisition.

### 2.3. Image Acquisition and Correction

To ensure the stable operation of the hyperspectral camera, the light source must be opened for the 30 min warm-up period before the experiment. The system parameters were set as follows: the HSI system was used to collect spectral data from the surface of the pears. The distance between the pear surface and the camera lens was 200 mm, the stage movement speed was 7.35 mm/s, and the camera exposure time was 8.50 ms to complete the hyperspectral image acquisition. The camera used a two-dimensional detector to capture the spectral data reflected by the sample through line-scanning, resulting in a collection of hyperspectral images with dimensions (x, y, λ), where x and y represent spatial dimensions (i.e., the number of rows and columns of pixels), representing the number of spectral bands. Due to a dark current and uneven illumination, the acquired hyperspectral images could not be directly used for subsequent data analysis. Therefore, black-and-white calibration had to be performed before the data acquisition. The Formula (1) for the black-and-white calibration was as follows:

(1) $$F=\frac{F_{0}-F_{b}}{F_{w}-F_{b}}$$

where *F* represents the corrected hyperspectral image, *F*_0_ denotes the original hyperspectral image, *F_w_* is the white image obtained using a standard white reference panel, and *F_b_* is the dark image. The corrected hyperspectral photos were used for subsequent spectral information extraction.

### 2.4. Quality Index Measurement

The firmness was evaluated with the fruit hardness tester (GY-4, Top Instrument, Hangzhou, China). The SSC was measured using the portable refractometer (PAL-1, Atago, Tokyo, Japan). The pH was determined with the pH meter (S210, Mettler-Toledo, Shanghai, China). The color difference (L*, a*, b*) of the fruit peel was evaluated using the colorimeter (CR-400, Konica Minolta, Japan). Additionally, the fruit maturity degree was assessed by measuring the index of absorbance difference (I_AD_) on the peel using a DA-Meter (University of Bologna, Italy).

### 2.5. Data Analysis

#### 2.5.1. Spectral Extraction

This experiment collected hyperspectral images of six pear varieties, each with 200 fruit samples, totaling 1200 images. ENVI 5.0 software was used to extract Regions of Interest (ROI) from the hyperspectral images of each sample. The average spectra of all pixels within the ROI were calculated to characterize the spectral information of each sample. Subsequently, these average spectra were used as input data for various spectral preprocessing methods and modeling techniques.

#### 2.5.2. Data Preprocessing

The hyperspectral camera was subject to light scattering and baseline drift during the data acquisition, necessitating the original spectral reflectance data preprocessing. In this study, we employed several preprocessing techniques: Standard Normal Variate (SNV) [17], Multiplicative Scatter Correction (MSC) [18], First Derivative (FD), and Second Derivative (SD) [19], as well as various combinations of these methods (SNV-FD, SNV-SD, FD-SNV, SD-SNV, MSC-FD, MSC-SD, FD-MSC, SD-MSC, FD-SD, SNV-MSC, and MSC-SNV) to preprocess the acquired raw spectral data. The spectral preprocessing was conducted using MATLAB 2012a (The Mathworks Inc., Natick, MA, USA) [20].

#### 2.5.3. Wavelength Selection

The successive projections algorithm (SPA) and competitive adaptive reweighted sampling (CARS) were employed to extract feature wavelengths from the spectral data, aiming to reduce the input variables and improve both the efficiency and predictive accuracy. SPA is a forward variable selection technique that iteratively selects variables based on their projection onto a hyperplane, minimizing multicollinearity by choosing those with minimal redundancy. This technique ensures that only the most relevant variables are retained, improving the model stability [21]. CARS, based on Monte Carlo sampling and partial least squares regression (PLSR), works by adaptively selecting variables with significant regression coefficients. It then refines the variable set through a reweighting process and selects the final subset using cross-validation error to optimize predictive performance. Both methods are designed to improve the robustness and accuracy of the model by reducing overfitting and enhancing the feature selection [22].

#### 2.5.4. Model Establishment and Evaluation

The data were randomly divided into training and prediction sets with a ratio of 3:1; this random division helps to improve the model’s generalization ability, enhances the reliability of the evaluation results, and contributes to the stability and accuracy of the model [23]. The LS-SVM is known for its strong generalization and robust regression capabilities, making it well-suited for this study [24]. The model performance was evaluated using *Rc* (Calibration Correlation Coefficient), *Rp* (Prediction Correlation Coefficient), *RMSEC* (Root Mean Square Error of Calibration), *RMSEP* (Root Mean Square Error of Prediction), and *RPD* (Ratio of Performance to Deviation). A correlation coefficient (R) close to 1, along with lower *RMSEC* and *RMSEP* values, indicates a well-fitting model and greater accuracy. An *RPD* value above 2.0 suggests predictive solid ability, while values between 1.5 and 2.0 are acceptable, and those below 1.5 indicate unsuitability for quantitative analysis [25].

In this study, four classification models were used to classify different varieties of pears, including Extreme Learning Machine (ELM) [26], Back Propagation Neural Network (BPNN) [27], Support Vector Machine (SVM) [28], and Random Forest (RF) [29]. The purpose of using these four models was to perform a comparative analysis in order to identify the most effective model for this classification task. By evaluating the performance of each model, we aimed to select the one that offered the highest classification accuracy.

## 3. Results and Discussion

### 3.1. Quality Parameters Analysis

Table 2 indicates notable differences in firmness, sugar content (SSC), pH values, color metrics (L*, a*, b*), and I_AD_ among six pear varieties. Significant disparities in firmness among various varieties provided a wealth of data variability, enriching the construction of a firmness regression model and enhancing the generalization capabilities of the model. The SSC was relatively uniform and stable across the varieties, allowing the model to converge more easily during training and generalize well. The peel color parameters, particularly the a* values, showed significant differences among the varieties. For instance, the a* values of the red pears ranged from 3.14 to 40.40, whereas other varieties, such as ‘Sucui No.1’ exhibited less variation. This was associated with the variations in the fruit appearance between different varieties. The color variations offered strong spectral responses in hyperspectral data. The I_AD_ had a wide numerical range that distinctly indicated variations in the degree of fruit maturity. However, the pH analysis of different pear varieties revealed a relatively narrow range.

### 3.2. Spectral Characteristics

**Figure 1:**
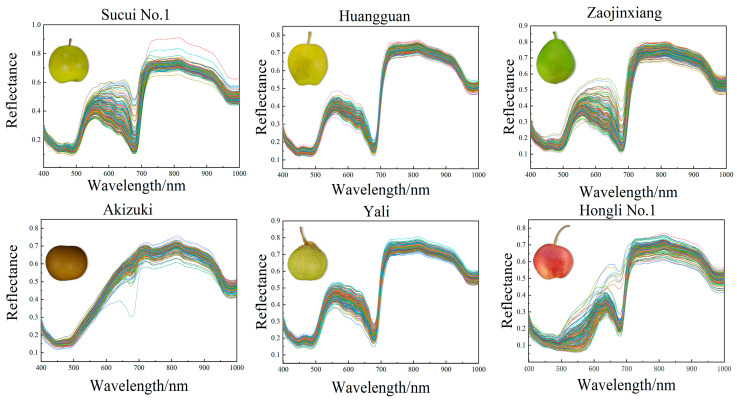
The reflectance spectra of the six different pear varieties.

The range of the spectra from 398 nm to 1004 nm was considered valid data in this study. It showed that the reflectance of the six pear varieties was similar in the visible and near-infrared spectral regions. Within the range of visible wavelength (from 500 nm to 600 nm), the reflectance of the six pear varieties showed a notable increase as the wavelength enlarged. The absorption peak at 680 nm was attributed to the presence of chlorophyll in the peel of the pear [30], while the peak variation at 920 nm was associated with water absorption [31]. All the pear varieties maintained high reflectance levels in the near-infrared region, but the extent of the changes exhibited differences. These differences provided the basis for pear variety identification, maturity degree assessment, and quality inspection (Figure 1).

### 3.3. Establishment of LS-SVM Model for Quality and Color Indicators

#### 3.3.1. Spectral Preprocessing

Using the example of predicting SSC for the Sucui No. 1 variety, the effective spectral range of 398–1004 nm was employed. Various preprocessing methods were applied, including FD, SD, SNV, MSC, and combinations of these techniques. These methods effectively reduced the spectral noise arising from the environmental factors, temperature fluctuations, and instrument errors, thereby enhancing the model robustness and reducing complexity. The results demonstrated that FD-SNV was the most effective preprocessing method (Figure S2 and Table S1). A comparative analysis was conducted among the models utilizing different preprocessing techniques, and a model was also established using the original spectra. FD-SNV emerged as the superior preprocessing method, with the most significant enhancement in prediction accuracy observed in the prediction set model. Following the FD-SNV preprocessing, the model values for *Rc*, *Rp*, and *RPD* showed a marked improvement. Notably, the *RPD* exceeded 2.5, indicating that the model possesses a strong predictive capability.

The quality analysis of the six pear varieties using the LS-SVM model (Table 2) showed that the sample range notably impacts the model’s predictive performance. For instance, the ‘Zaojinxiang’ and ‘Hongli No.1’ exhibited a broad hardness range with substantial differences between the highest and lowest values in hardness prediction. This enables the model to leverage variations among different hardness levels effectively (*RPD* value > 2.5), demonstrating high predictive accuracy (Table 3). In addition, the prediction accuracy for other parameters like SSC, pH, L*, a*, b*, and I_AD_ was likewise significantly influenced by the sample range. From the SSC analysis, ‘Sucui No.1’, ‘Huangguan’, and ‘Yali’ showed high prediction accuracy in SSC with the *RPD* values exceeding 2. The *RPD* value of the ‘Huangguan’ was 5.4, suggesting that the model had an excellent predictive capability for this parameter. This could be due to the wide range of SSC values that enabled the model to capture the precise correlation between the spectral variations and SSC. It has been proposed that the more significant the sample variability, the more effectively the model could capture critical features related to quality parameters through hyperspectral data, thus enhancing the prediction accuracy [32].

The prediction of the pH was generally inaccurate in the six pear varieties, with the *RPD* values mostly ranging from 0.7 to 1.0. It might be that HSI obtained characteristics through physical changes of the surface’s reflectance and absorption, while the pH value was more affected by internal components, so these changes were very weak in the spectral reflectance characteristics and it was difficult to form effective discriminant signals. According to previous studies, the near-infrared spectroscopy had limitations in predicting the acidity of the fruits and vegetables. In most fruits and vegetables, the acid concentrations were typically much lower than the sugar concentrations and might be too low to influence the near-infrared spectroscopy significantly [33].

Regarding the color of the fruit (L*, a*, b*), ‘Zaojinxiang’ exhibited the best predictive performance, and the *RPD* values of L* and a* were 2.9 and 3.0, respectively. This suggested that for the sample range and different color degrees, using the LS-SVM model could mean a better relationship between the spectra and color. This finding was similar to the result that the color prediction by HSI was closely related to the range of color differences among the samples, with more extensive ranges contributing to enhance the prediction accuracy [34].

Regarding the I_AD_ prediction, the *RPD* values of all the pear varieties exceeded 2.0, indicating that the LS-SVM model could accurately predict the I_AD_. This result could also be ascribed to the wide range of I_AD_ values [35].

#### 3.3.2. Feature Wavelength Selection Results

To identify the method for selecting the feature wavelength, the initial spectra were individually processed with FD-SNV, and then were screened using CARS and SPA. Subsequently, the filtered characteristic spectra as the input data, and the measured SSC of ‘Sucui No.1’ as the output data were used to establish the LS-SVM prediction model, and the results were comparatively analyzed.

Based on the predicted result for the SSC of ‘Sucui No.1’, the CARS significantly reduced the redundancy data and effectively enhanced the prediction accuracy of the model (Figure S3 and Table S1). Following the CARS screening, the number of variables was reduced from 448 to 44. Compared to the models without a variable selection, the CARS model’s *RPD* value on the prediction set increased from 2.8 to 2.9, and the correlation coefficient was slightly improved, while the *RMSEP* decreased. Although the CARS efficiently reduced the redundancy data and simplified the model, the degree of improvement was relatively limited. It might be the result of the complexity of the hyperspectral data, which caused the CARS to be unable to comprehensively obtain all the information strongly related to the target quality parameters. It was similar to the sweet potato study [36].

#### 3.3.3. Modeling Analysis Based on Feature Wavelength

Following the optimal data preprocessing method FD-SNV, CARS was employed to select the wavelengths, which served as input data to establish the LS-SVM models for predicting various parameters of the six pear varieties (Table 4).

By applying the FD-SNV preprocessing to the initial spectral data and integrating it with the CARS wavelength selection, the I_AD_ prediction performance for the ‘Sucui No.1’ increased from the *RPD* value from 3.5 to 3.9. The *RPD* value for the hardness prediction of the ‘Zaojinxiang’ improved from 2.8 to 3.1, the *RPD* value for the color prediction of b* was enhanced from 1.6 to 2.3, and the *RPD* value for the I_AD_ prediction improved from 3.4 to 4.0. The *RPD* value for the SSC prediction of the ‘Akizuki’ improved from 1.9 to 2.0. In the ‘Yali’, the *RPD* value for the SSC increased from 2.8 to 3.3, and L* from 1.9 to 2.1. All of the parameter predictions affected for the ‘Hongli No.1’ were enhanced.

In summary, the model has been optimized by the data preprocessing and wavelength screening, but it was limited. Although the screening of the feature wavelengths reduced the number of variables and simplified the model, in certain instances, such as predicting L*, a*, and b*, the *RPD* values did not significantly increase. Further analysis showed that the influence of the sample range on the model accuracy was more pronounced. The number of samples and data diversity constrained the model prediction accuracy for single-variety samples. To improve the stability of the model and the predictive accuracy, it was necessary to further integrate the sample data from the six pear varieties.

#### 3.3.4. Development of a Joint Model to Predict Different Indicators for the Six Pear Varieties

To enhance the generalization capacity of the model, the data of the six pear varieties were consolidated in this work. Considering that FD-SNV and CARS could effectively improve the *RPD* value, the consolidated data of the six pear varieties were preprocessed with FD-SNV firstly (output data), selecting characteristic wavelengths via CARS as input data, and then the LS-SVM model was reestablished.

The *RPD* value for firmness increased to 3.4 by reestablishing the LS-SVM model. This might be the result of the increased data and variability of samples. Although the *RPD* values for SSC (Table 5) decreased comparing to the result in the single pear variety (Table 4), it was above 2.0. Therefore, the model still worked well. Different from SSC, the *RPD* value for pH, L*, a*, b*, and I_AD_ value significantly increased by reestablishing the LS-SVM model (Table 5), demonstrating a notable enhancement in the model’s predictive performance. Moreover, the reestablished LS-SVM showing higher *Rc* and *Rp* values but lower *RMSEC* and *RMSEP* values, indicated that it enhanced the prediction accuracy and generalization capabilities. These results were consistent with those of previous studies that sample diversity enhanced the model stability, making it more effective in practical applications [37]. Many studies have been focused on the quality detection of a single fruit variety, while this work significantly improved the stability and applicability of the model by expanding the sample diversity and quantity.

#### 3.3.5. The Development of a Classification Model for the Six Pear Varieties

**Figure 2:**
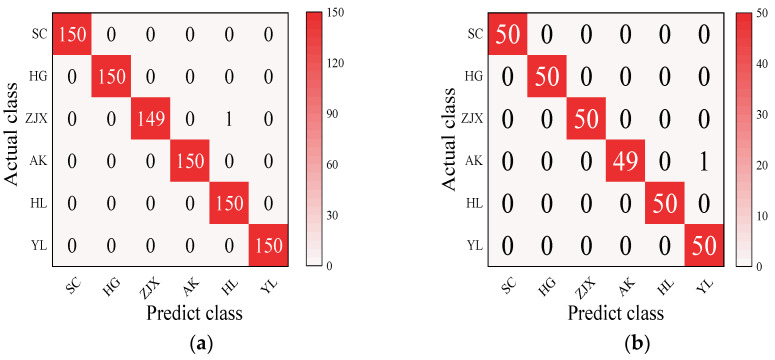
BP neural network classification model: (**a**) Calibration set; (**b**) Prediction set. These abbreviations are detailed as follows: Sucui No.1: SC; Huangguan: HG; Zaojinxiang: ZJX; Akizuki: AK; Hongli No.1: HL; Yali: YL. The same form is used in Figure 3, Figure 4 and Figure 5.

**Figure 3:**
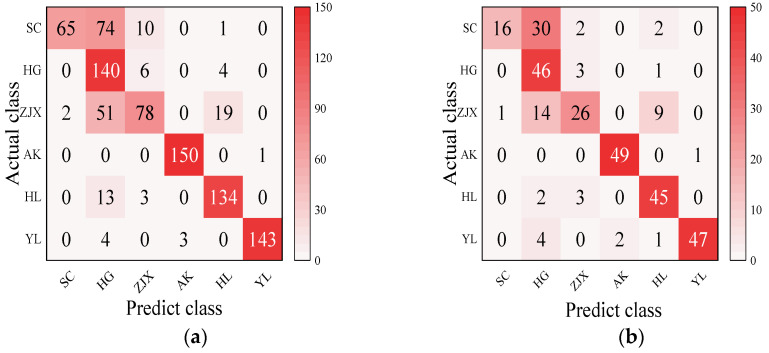
SVM classification model: (**a**) Calibration set; (**b**) Prediction set.

**Figure 4:**
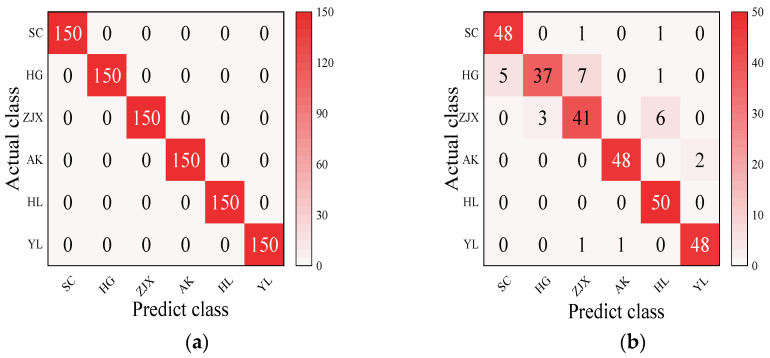
RF classification model: (**a**) Calibration set; (**b**) Prediction set.

**Figure 5:**
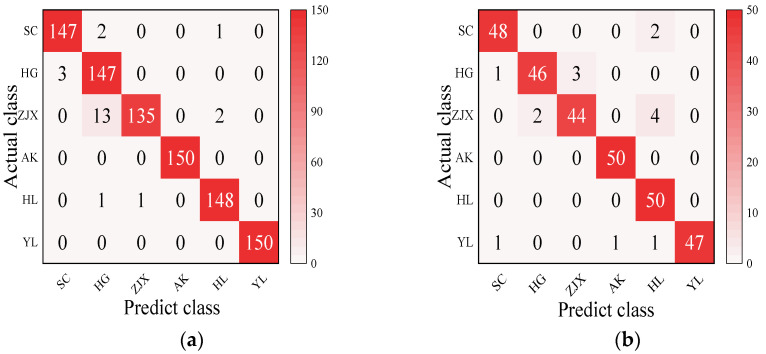
ELM classification model: (**a**) Calibration set; (**b**) Prediction set.

Four models—BP neural network, SVM, RF, and ELM—were evaluated in the classification model analysis of the six pear varieties (Table 6). The BP neural network performed exceptionally well on all the varieties, with the prediction accuracy rates close to 100%, suggesting its strong ability to handle nonlinear relationships in spectral data [38] (Figure 2). SVM performed well on the pear varieties like ‘Hongli No.1’ (97.9%) and ‘Akizuki’ (96.1%), but its accuracy was only 50% for the ‘Huangguan’ variety, indicating some limitations in the classification. It may struggle with complex classification tasks and is highly dependent on parameter tuning [39] (Figure 3). The RF model also performed well on most varieties, with the prediction accuracy reaching 100% for some. However, its performance slightly declined for the ‘Sucui No.1’ and ‘Huangguan’, which may be related to the random generation of trees in the random forest model, leading to a poorer performance in varieties with a significant feature overlap [40] (Figure 4). The ELM performed well overall, but its prediction accuracy for the ‘Yali’ variety was only 87.7%. This may be due to the random initialization of weights in ELM, which affects its generalization ability when dealing with noisy or imbalanced data [41] (Figure 5).

In summary, the BP neural networks and random forests had more excellent stability in the classification performance. At the same time, the SVM and ELM models could require further optimization or feature engineering to improve their classification effectiveness.

## 4. Conclusions

This study integrated HSI technology with machine learning models to perform a non-destructive quality assessment of six pear varieties, confirming the effectiveness and potential application in fruit quality detection. The results indicated that applying suitable spectral preprocessing FD-SNV and feature wavelength selection CARS greatly improved the prediction accuracy and robustness of the model. Integrating data from multiple pear varieties further increased the model’s generalization capability. The model showed extreme precision and stability, particularly on predicting the SSC and maturity degree. This offers a practical approach for achieving rapid and precise evaluation of pear quality.

On this basis, future research could focus on expanding the model to include a wider variety of fruits and explore the use of more advanced machine learning techniques, such as deep learning, to improve prediction accuracy. Additionally, developing real-time, on-field quality evaluation systems could further enhance the practicality of this approach.

## Figures and Tables

**Table 1:** Material information.

Variety	Collection Date	Origin	Coordinates
Sucui No.1	10 July 2023	Ningjin, Hebei	N: 37.77°, E: 114.53°
Huanggua	3 August 2023	Xinji, Hebei	N: 37.28°, E: 115.11°
Zaojinxiang	11 August 2023	Fucheng, Hebei	N: 38.01°, E: 115.96°
Akizuki	5 September 2023	Zhaoxian, Hebei	N: 37.76°, E: 114.96°
Yali	19 September 2023	Zhaoxian, Hebei	N: 37.76°, E: 114.96°
Hongli No.1	19 October 2023	Shahe, Hebei	N: 37.80°, E: 114.99°

**Table 2:** Quality parameters of six pear varieties.

Variety	Parameter	Min	Max	Mean	Standard Deviation
Sucui	Firmness (N)	37.53	93.16	60.18	11.38
No.1	SSC (%)	9.65	14.45	12.66	0.95
	pH	4.99	5.82	5.32	0.14
	L*	53.08	73.65	65.72	3.32
	a*	−19.19	−1.68	14.96	3.44
	b*	47.76	58.43	53.99	1.84
	I_AD_	0	1.7	1.01	0.38
Huangguan	Firmness (N)	57.72	91.73	72.08	7.02
	SSC (%)	9.40	12.95	11.19	0.79
	pH	4.12	4.82	4.56	0.11
	L*	52.20	69.93	65.64	2.07
	a*	−17.13	−11.67	−14.47	1.00
	b*	34.01	47.37	41.56	1.77
	I_AD_	0.83	1.61	1.19	0.15
Zaojinxiang	Firmness (N)	25.97	214.42	123.95	49.29
	SSC (%)	7.75	14.90	11.85	1.18
	pH	4.02	4.82	4.39	0.16
	L*	57.19	76.38	64.82	3.77
	a*	−19.53	−4.39	−14.46	3.53
	b*	35.74	48.80	42.48	2.79
	I_AD_	0.22	2.00	1.43	0.44
Akizuki	Firmness (N)	38.51	77.62	56.46	8.51
	SSC (%)	8.05	15.35	11.70	1.71
	pH	4.73	5.17	4.94	0.09
	L*	57.75	64.73	61.75	1.59
	a*	−8.61	−1.39	−5.87	1.10
	b*	30.78	39.17	34.21	1.57
	I_AD_	0.00	0.55	0.02	0.05
Yali	Firmness (N)	55.76	77.13	64.38	4.49
	SSC (%)	8.20	14.30	11.53	0.94
	pH	4.13	4.88	4.49	0.15
	L*	63.84	76.97	70.78	2.43
	a*	−18.07	−10.04	−14.41	1.49
	b*	38.49	48.08	42.61	1.68
	I_AD_	0.50	1.40	0.95	0.20
Hongli	Firmness (N)	74.28	187.87	120.35	27.62
No.1	SSC (%)	8.95	16.50	11.72	1.47
	pH	4.08	4.88	4.57	0.14
	L*	36.38	64.81	46.12	5.48
	a*	3.14	40.40	−21.95	6.36
	b*	15.66	37.92	23.68	4.30
	I_AD_	0.04	1.54	0.91	0.22

**Table 3:** Prediction of different quality parameters of six varieties of pears.

Variety	Parameter	Calibration Set		Prediction Set		*RPD*
		*Rc*	*RMSEC*	*Rp*	*RMSEP*	
Sucui	Firmness (N)	0.951	0.302	0.839	0.496	1.7
No. 1	SSC (%)	0.940	0.312	0.935	0.373	2.8
	pH	0.743	0.088	0.579	0.123	0.7
	L*	0.888	1.539	0.875	1.594	1.8
	a*	0.925	1.257	0.897	1.714	2.1
	b*	0.893	1.622	0.903	1.410	2.2
	I_AD_	0.959	0.105	0.964	0.104	3.5
Huangguan	Firmness (N)	0.741	5.018	0.462	5.217	0.9
	SSC (%)	0.985	0.142	0.984	0.156	5.4
	pH	0.737	0.074	0.474	0.096	0.7
	L*	0.782	1.348	0.743	1.219	1.2
	a*	0.994	0.163	0.733	0.601	1.3
	b*	0.756	1.213	0.622	1.231	1.0
	I_AD_	0.947	0.048	0.939	0.052	2.8
Zaojinxiang	Firmness (N)	0.945	16.609	0.946	14.559	2.8
	SSC (%)	0.964	0.303	0.934	0.430	2.4
	pH	0.825	0.104	0.475	0.153	0.5
	L*	0.939	1.360	0.946	1.013	2.9
	a*	0.926	1.333	0.958	1.051	3.0
	b*	0.903	1.199	0.848	1.506	1.6
	I_AD_	0.956	0.129	0.969	0.115	3.4
Akizuki	Firmness (N)	0.788	5.667	0.784	4.705	1.1
	SSC (%)	0.928	0.643	0.891	0.726	1.9
	pH	0.862	0.048	0.622	0.062	0.9
	L*	0.842	0.845	0.872	0.847	1.7
	a*	0.851	0.565	0.892	0.558	1.7
	b*	0.817	0.928	0.839	0.832	1.5
	I_AD_	0.920	0.023	0.884	0.021	2.0
Yali	Firmness (N)	0.623	3.585	0.533	3.911	0.6
	SSC (%)	0.954	0.267	0.948	0.361	2.8
	pH	0.753	0.133	0.559	0.135	1.0
	L*	0.899	1.066	0.871	1.120	1.9
	a*	0.819	0.862	0.827	0.810	1.6
	b*	0.842	0.965	0.813	0.801	1.4
	I_AD_	0.945	0.066	0.942	0.066	2.7
Hongli	Firmness (N)	0.945	8.903	0.926	9.025	2.6
No.1	SSC (%)	0.989	0.218	0.973	0.386	3.7
	pH	0.779	0.094	0.770	0.094	0.8
	L*	0.834	3.367	0.826	2.816	1.6
	a*	0.751	4.182	0.830	3.798	1.3
	b*	0.902	1.855	0.815	2.510	1.5
	I_AD_	0.927	0.086	0.922	0.10	2.5

**Table 4:** The prediction results for the quality parameters of the six pear varieties by LS-SVM model.

Variety	Parameter	Calibration		Prediction		*RPD*
		*R_c_*	*RMSEC*	*R_p_*	*RMSEP*	
Sucui	Firmness (N)	0.783	6.967	0.813	7.083	1.6
No. 1	SSC (%)	0.929	0.337	0.940	0.364	2.9
	pH	0.764	0.089	0.687	0.10	0.8
	L*	0.860	1.683	0.825	1.837	1.7
	a*	0.901	1.454	0.891	1.716	2.1
	b*	0.904	1.472	0.903	1.560	2.1
	I_AD_	0.965	0.097	0.967	0.094	3.9
Huangguan	Firmness (N)	0.718	4.934	0.60	5.763	1.0
	SSC (%)	0.930	0.279	0.951	0.264	3.0
	pH	0.718	0.075	0.604	0.084	0.7
	L*	0.80	1.170	0.820	1.194	1.7
	a*	0.814	0.583	0.749	0.676	1.3
	b*	0.904	1.019	0.903	1.167	1.1
	I_AD_	0.965	0.097	0.967	0.093	3.9
Zaojinxiang	Firmness (N)	0.947	15.823	0.947	16.609	3.1
	SSC (%)	0.961	0.322	0.911	0.466	2.5
	pH	0.718	0.120	0.604	0.122	0.7
	L*	0.946	1.249	0.944	1.178	3.0
	a*	0.921	1.315	0.749	1.234	3.1
	b*	0.881	1.327	0.905	1.222	2.3
	I_AD_	0.944	0.148	0.967	0.106	4.1
Akizuki	Firmness (N)	0.886	4.083	0.711	6.322	1.4
	SSC (%)	0.909	0.731	0.895	0.712	2.0
	pH	0.866	0.047	0.626	0.058	1.0
	L*	0.817	0.921	0.845	0.830	1.6
	a*	0.856	0.577	0.856	0.556	1.8
	b*	0.863	0.808	0.811	0.909	1.5
	I_AD_	0.907	.024	0.872	0.018	2.0
Yali	Firmness (N)	0.886	4.083	0.711	3.445	0.8
	SSC (%)	0.990	0.271	0.956	0.326	3.3
	pH	0.697	0.141	0.621	0.121	1.1
	L*	0.880	1.167	0.892	1.039	2.1
	a*	0.850	0.773	0.867	0.762	1.7
	b*	0.810	0.988	0.816	0.975	1.6
	I_AD_	0.957	0.057	0.967	0.078	2.7
Hongli	Firmness (N)	0.950	8.367	0.934	10.765	2.7
No.1	SSC (%)	0.977	0.305	0.953	0.430	3.8
	pH	0.697	0.141	0.621	0.122	0.9
	L*	0.811	3.325	0.836	3.441	1.7
	a*	0.767	3.899	0.767	3.890	1.3
	b*	0.862	2.259	0.823	2.164	1.6
	I_AD_	0.926	0.086	0.935	0.078	2.7

**Table 5:** Prediction results of quality parameters of six varieties of pear by LS-SVM model.

Parameter	No. of Original Samples	Selected Variables	Calibration		Prediction		*RPD*
			*R_c_*	*RMSEC*	*R_p_*	*RMSEP*	
Firmness (N)	1200	31	0.959	10.864	0.959	10.805	3.4
SSC (%)	1200	49	0.901	0.556	0.915	0.584	2.2
pH	1200	53	0.950	0.107	0.929	0.135	2.6
L*	1200	53	0.980	1.680	0.974	1.919	4.3
a*	1200	30	0.991	1.923	0.989	2.146	6.9
b*	1200	41	0.988	1.499	0.974	2.031	4.4
I_AD_	1200	27	0.981	0.099	0.987	0.085	6.0

**Table 6:** Classification model for different pear varieties.

Model Category	Parameter	Calibration		Prediction	
		Samples	Accuracy (%)	Samples	Accuracy (%)
**BP Neural Network**	Sucui. No 1	150	100	50	100
	Huangguan	150	100	50	100
	Zaojinxiang	150	100	50	100
	Akizuki	150	100	50	100
	Yali	150	99.3	50	100
	Hongli	150	100	50	98.0
**SVM**	Sucui. No 1	150	97.0	50	94.1
	Huangguan	150	49.6	50	50.0
	Zaojinxiang	150	80.4	50	76.5
	Akizuki	150	98.0	50	96.1
	Yali	150	84.8	50	77.6
	Hongli	150	100	50	97.9
**RF**	Sucui. No 1	150	100	50	90.6
	Huangguan	150	100	50	50.0
	Zaojinxiang	150	100	50	76.5
	Akizuki	150	100	50	96.1
	Yali	150	100	50	77.6
	Hongli	150	100	50	97.9
**ELM**	Sucui. No 1	150	98.0	50	96.0
	Huangguan	150	90.2	50	95.8
	Zaojinxiang	150	99.3	50	93.6
	Akizuki	150	100	50	98.0
	Yali	150	98.0	50	87.7
	Hongli	150	100	50	100.0

## Data Availability

The original contributions presented in the study are included in the article/Supplementary Materials, further inquiries can be directed to the corresponding authors.

## Supplementary Materials

The following supporting information can be downloaded at: https://www.mdpi.com/article/10.3390/foods13233956/s1. Figure S1: Vis‐NIR hyperspectral imaging system; Figure S2: The reflectance spectra of Sucui No.1 (a) raw spectrum; (b) FD-SNV; Figure S3: The reflectance spectra of Sucui No.1 (a) CARS (b) SPA; Figure S4: Six pear quality regression models; Figure S5: The ROI figure of Akizuki; Table S1: Performance of LS-SVM models for SSC of ‘Sucui No.1’; Table S2: The prediction results of SSC of SuCui by LS-SVM models developed by using the influential variables.
