# Supplementary material for: Detection of Pear Quality Using Hyperspectral Imaging Technology and Machine Learning Analysis
Source: Foods. 2024 Dec 8;13(23):3956. doi: 10.3390/foods13233956 (PMC11640658; doi:10.3390/foods13233956)
Supplement: Supplementary file 1 [file foods-13-03956-s001.zip › foods-3343559-supplementary.pdf]

Detection of Pear Quality Using Hyperspectral Imaging Technology and Machine Learning Analysis

Zishen Zhang [1,2,3], Hong Cheng [2,3], Meiyu Chen [2,3,4], Lixin Zhang [2,3], Yudou Cheng [2,3], Wenjuan Geng [1,*] and Junfeng Guan [2,3,*]

[1] College of Horticulture, Xinjiang Agricultural University, Urumqi 830052, China; zhangzishen2@163.com

[2] Institute of Biotechnology and Food Science, Hebei Academy of Agricultural and Forestry Sciences, Shijiazhuang 050051, China; chenghonghappyok@163.com (H.C.); cmy20000128@126.com (M.C.); feiliuzhao@163.com (L.Z.); chengyudouyn@163.com (Y.C.)

[3] Hebei Key Laboratory of Plant Genetic Engineering, Shijiazhuang 050051, China

[4] College of Life Science and Food Engineering, Hebei University of Engineering, Handan 056000, China

* Correspondence: gwj0526@xjau.edu.cn (W.G.); guanjf@haafs.org (J.G.)

Supplementary Material

The hyperspectral imaging system comprises a high spectrograph, a CCD camera, two tungsten halogen lamps, a moving platform, and a computer with image acquisition software.

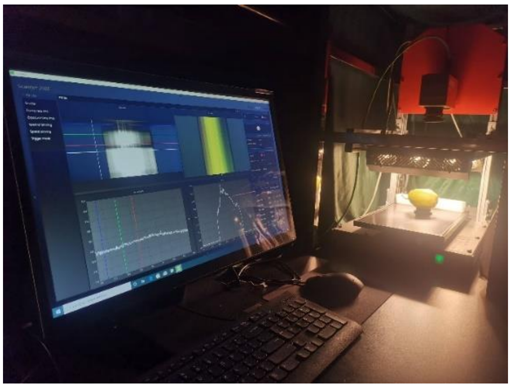

Figure S1. Vis-NIR hyperspectral imaging system.

The evaluation of the LS-SVM model results for SSC content of 'Sucui No.1' under different spectral preprocessing methods are shown in Table S1. Spectral prediction models pre-processed with FD-SNV performed best are shown in Figure S2

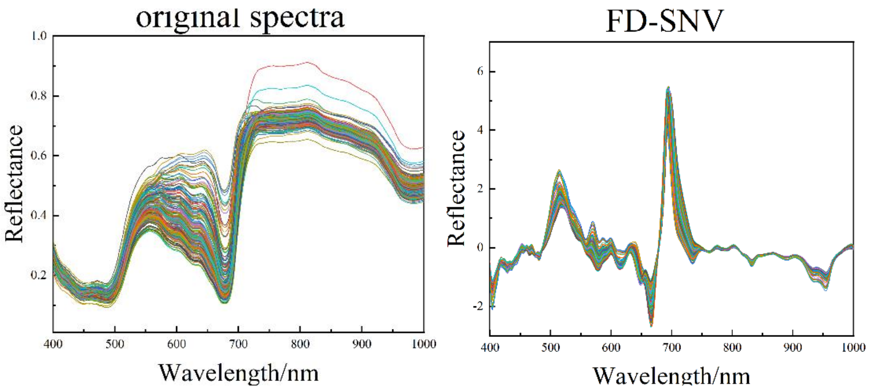

(**a**) (**b**)

Figure S2. The reflectance spectra of Sucui No 1(**a**) raw spectrum; (**b**) FD-SNV

Table S1. Performance of LS-SVM models for SSC of 'Sucui No.1'.

**Preprocessing**	**Calibration set**		**Prediction set**		***RPD***
	R_c	*RMSEC*/°**Brix**	R_p	*RMSEP*/°**Brix**	
Original	0.951	0.302	0.839	0.496	1.7
SNV	0.957	0.289	0.828	0.499	1.6
MSC	0.941	0.324	0.916	0.414	2.2
FD	0.951	0.308	0.66	0.436	1.7
SD	0.967	0.276	0.815	0.480	1.6
SNV+FD	0.949	0.317	0.850	0.505	1.8
FD+SNV	0.940	0.312	0.935	0.373	2.8
SNV+SD	0.981	0.205	0.829	0.532	1.7
SD+SNV	0.946	0.313	0.931	0.352	2.5
MSC+FD	0.923	0.368	0.829	0.382	2.5
FD+MSC	0.931	0.352	0.934	0.344	2.6
MSC+SD	0.936	0.343	0.843	0.364	2.4
SD+MSC	0.943	0.318	0.867	0.364	2.4

The processed spectra were then screened using CARS and SPA. The selected feature spectra served as inputs, and the measured soluble solids content (SSC) of 'Sucui No. 1' was used as the output to establish an LS-SVM predictive model. The results were compared and analyzed. The modeling results using different feature wavelength selection methods are shown in Table S2.

Table S2. The prediction results of SSC of SuCui by LS-SVM models developed by using the influential variables

Wavelength selection	No. of samples	calibration		prediction		*RPD*
		R_c	*RMSEC*	R_p	*RMSEP*	
None	448	0.940	0.312	0.935	0.373	2.8
CARS	44	0.929	0.337	0.940	0.364	2.9
SPA	7	0.765	0.589	0.780	0.663	1.2

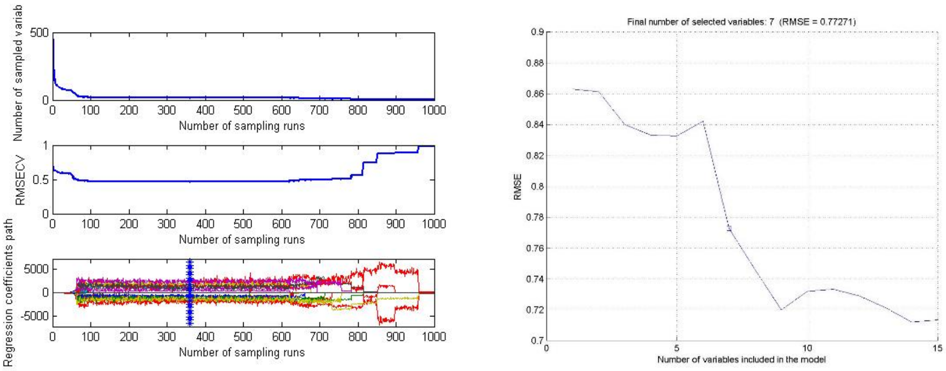

(**a**) (**b**)

Figure S3**.** The reflectance spectra of Sucui No 1(**a**) CARS (**b**) SPA

The optimal preprocessing method, FD-SNV, was applied. CARS was used for feature wavelength selection to build LS-SVM models to predict the quality parameters of the six pear cultivars shown.

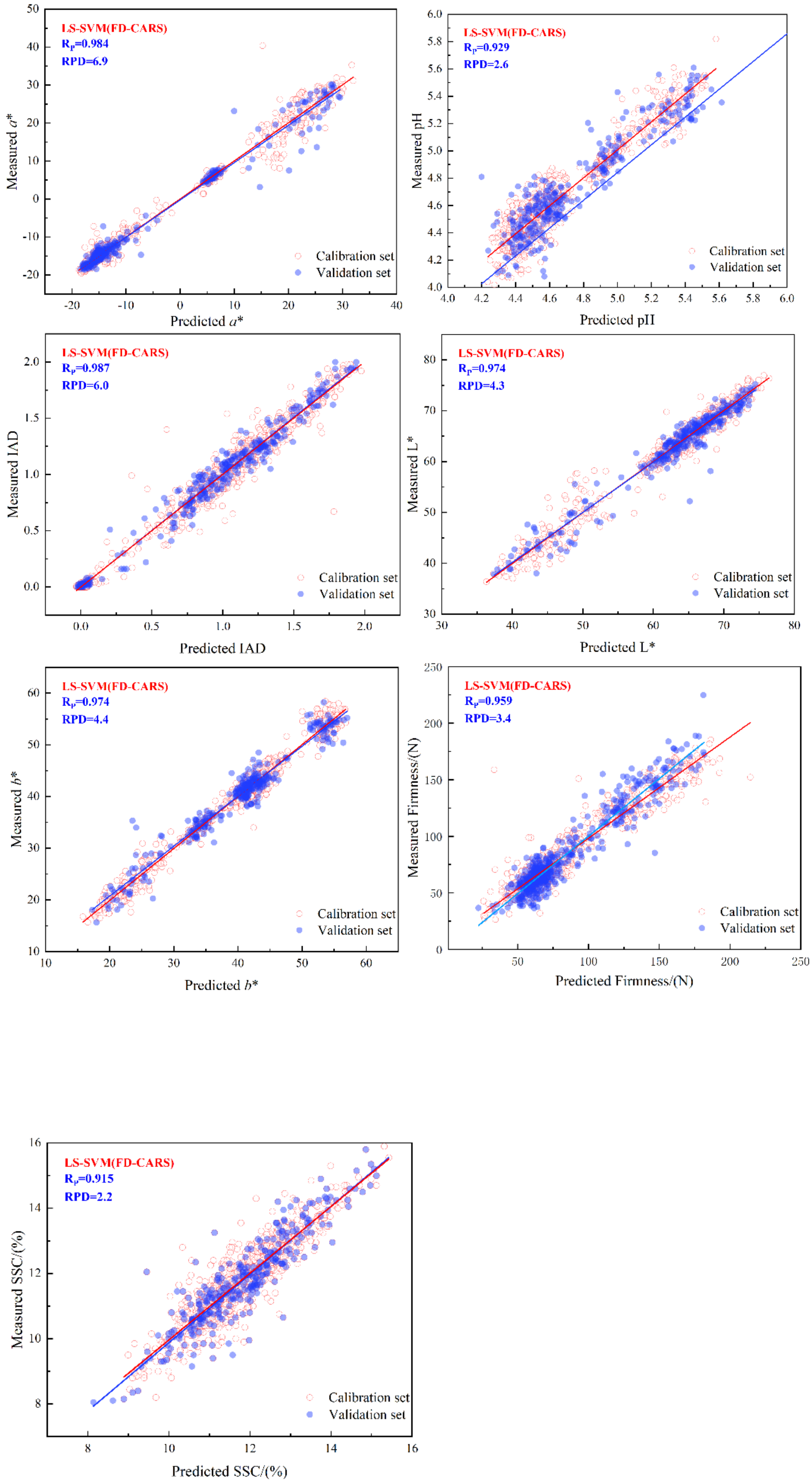

Figure S4. Six pear quality regression models

This experiment collected hyperspectral images of six pear varieties, each with 200 fruit samples, totaling 1,200 images. ENVI 5.0 software was used to extract Regions of Interest (ROI) from the hyperspectral images of each sample.

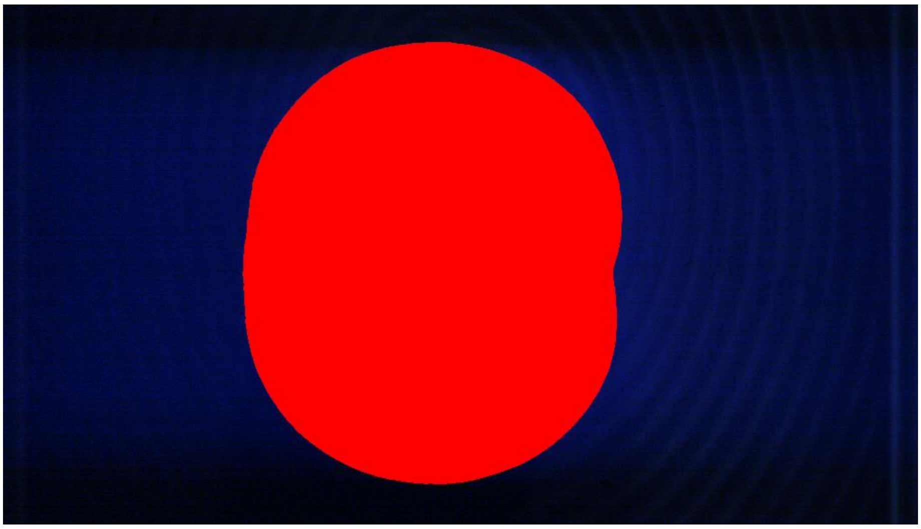

Figure S5**.** The ROI figure of Akizuki
